# Integrative metabolomic and transcriptomic profiling deciphers flavonoid biosynthesis of bulbs in *Lilium brownii* var. *Viridulum*

**DOI:** 10.1038/s41598-026-43274-5

**Published:** 2026-03-17

**Authors:** Pu-Tao Wang, Ya-Jie Xue, Fan Liu, Tao Chang, You Qin, Qin-Fang Zheng

**Affiliations:** 1 Key Laboratory of Dong Medical Research of Hunan Province, Hunan University of Medicine, Huaihua, 418000 People’s Republic of China; 2 Institute of Chinese Materia Medica, Hunan Academy of Chinese Medicine, Changsha, 410013 People’s Republic of China; 3 Biomedical Research Insititute, Hunan University of Medicine, Huaihua, 418000 People’s Republic of China; 4 Key Laboratory of Information Cultivation Research of Huaihua City, Hunan University of Medicine, Huaihua, 418000 People’s Republic of China

**Keywords:** Flavonoid, Metabolome, Transcriptome, Bulbs of *Lilium brownii* var. *viridulum*, Biochemistry, Biotechnology, Molecular biology, Plant sciences

## Abstract

*Lilium brownii* var. *viridulum* is rich in flavonoids, which offer antioxidant, anti-inflammatory, antitumor, and immunomodulatory effects. Recently, a novel, more resistant variant called *L. brownii* var. *viridulum* (Xuefeng, xf) was identified through individual plant selection. However, the metabolic and transcriptional profiles of xf, particularly concerning flavonoid metabolism, have not yet been compared to those of the traditional variant (Longya, ly). To address this gap, we performed an integrated metabolomic and transcriptomic analysis to systematically compare flavonoid metabolism between ly and xf. Our metabolomic analysis identified 85 flavonoids that are significantly enriched in the xf variety. In the transcriptomic analysis, we identified 59 differentially expressed enzyme genes, with expression levels in xf being significantly higher than those in ly, consistent with the metabolite profile. By combining transcriptome and metabolome, we identified one MYB transcription factor (Cluster-6560.0) and four bHLH transcription factors (Cluster-22934.6, Cluster-22934.10, Cluster-24120.2, and Cluster-24120.13) linked to anthocyanin synthesis. The co-expression of these identified genes supports the enrichment of flavonoids in xf, and this coordinated expression pattern of key enzymes was further confirmed via real-time quantitative PCR (qRT-PCR). In conclusion, this study enhances our understanding of the molecular mechanisms underlying flavonoid biosynthesis and provides a strong foundation for the genetic breeding of lilies.

**Supplementary Information:**

The online version contains supplementary material available at 10.1038/s41598-026-43274-5.

## Introduction

Lilium, a member of the genus Lilium within the family Liliaceae, is a well-known medicinal and edible plant with deep cultural and therapeutic importance. As a traditional nutraceutical, its primary edible and pharmacologically active part is the bulb. Pharmacological studies have confirmed that its bioactive components mainly include saponins, polysaccharides, flavonoids, and alkaloids^1^. Lilium has therapeutic uses, including relieving cough and asthma, lowering blood glucose, exhibiting antitumor activity, improving sleep quality, enhancing immune function, and aiding in the prevention of Alzheimer’s disease^1^. In cooking, it is used inrefined Chinese dishes: during summer, it is simmered into lily bulb soup or porridge to dispel heat, clear summer-heat, and moisten the lungs, while other classic preparations include sautéed lily bulbs with celery and lily bulbs stir-fried with sliced pork. *L. brownii* var. *viridulum*, known as “Longya” in Chinese, is a medicinal lily listed in the Pharmacopoeia of the People’s Republic of China and serves as a key economic crop in Shaoyang County, Hunan Province^2,3^. Rich in phenolic compounds, flavonoids, alkaloids, and polysaccharides, this plant demonstrates strong antioxidant, anti-inflammatory, anti-tumor, and immunomodulatory properties, making it promising for preventing and treating chronic diseases. Flavonoid compounds are a primary focus of current research^4,5^. The Longya lily, cultivated in Shaoyang—particularly Longhui County—and surrounding areas like Xiangtan, Hunan, is recognized as one of the province’s “Xiangjiuwei” elite medicinal herbs due to its unique pharmacological traits. The single-plant line “Xuefeng” (xf) was sourced from the Xuefeng Mountains and officially identified as *L. brownii* var. *viridulum* by Associate Researcher Liu Hao of the Hunan Academy of Chinese Medicine. Compared to the traditional ‘Longya’ (ly) cultivar, this line exhibits significantly enhanced disease resistance (Fig. S1). The metabolic differences between these two varieties of Longya have yet to be fully clarified. Understanding these differences is crucial for cultivar authentication, quality evaluation, and mechanistic research.

Longya bulbs are rich in phenolics, particularly flavonoids with strong antioxidant properties, making systematic comparison of their metabolite profiles across cultivars or treatments timely and valuable for identifying superior germplasm^7–9^. Since flavonoids cannot be synthesized endogenously by humans, this plant-derived secondary metabolites are essential dietary components for maintaining overall health^10^. Integrating metabolomic data with transcriptomic or genomic analyses will enable the identification of the key genes and regulatory factors involved in flavonoid biosynthesis in *L. brownii* var. *viridulum*^11^. Elucidating these molecular determinants is essential for efficiently screening of superior germplasm and developing targeted breeding programs for functional lily varieties.

In plant cells, flavonoid biosynthesis primarily proceeds via the phenylpropanoid pathway, which begins in the cytoplasm through phenylalanine metabolism^12^. This intricate biochemical process is regulated by several rate-limiting enzymes, such as phenylalanine ammonia-lyase (PAL), cinnamate 4-hydroxylase (C4H), chalcone synthase (CHS), and chalcone isomerase (CHI). Subsequent modification steps involve flavonoid 3-hydroxylase (F3H), flavonoid 3′,5′-hydroxylase (F3′5′H), flavonol synthase (FLS), and dihydroflavonol 4-reductase (DFR)^12^.

Flavonoid biosynthetic genes are tightly regulated at the transcriptional level by several regulatory proteins, notably MYB, bHLH and WD40, which together control the expression of flavonoid-related genes^13,14^. These three transcription factors can act independently or cooperatively to form the MYB-bHLH-WD40 (MBW) transcriptional complex^15^. Significantly, these transcription factors have been demonstrated to synergistically activate numerous genes responsible for anthocyanin production in across angiosperms^16^. In the MBW complex, the MYB transcription factor acts as the central regulator of anthocyanin biosynthesis in plants, controlling both its intensity and pattern^16^. Additionally, individual MYB and bHLH transcription factors, such as SbMYB3 (*Scutellaria baicalensis*)^17^, PbMYB211 (*Phoebe bournei*)^18^, and SlbHLH95 (*Solanum lycopersicum*)^19^, regulate flavonoid biosynthesis. While prior studies have identified candidate genes related to flavonoid synthesis in lilies^20^, there is limited information available on flavonoid synthesis-related genes in *L. brownii* var. *viridulum*.

Integrated transcriptomic and metabolomic analyses has emerged as a powerful approach for elucidating the biosynthetic pathways and regulatory networks of bioactive constituents in medicinal plants^21,22^. In this study, combined metabolomic and transcriptomic analyses were used to dissect the biosynthetic mechanisms of flavonoids in two cultivars of *L. brownii* var. *viridulum* (ly and xf). Key genes implicated in these pathways were identified and their functions were preliminarily functionally validated. These findings lay the groundwork for rationally exploiting the edible and medicinal value of *L. brownii* var. *viridulum* through molecular breeding and metabolic engineering, and provide new insights into quality improvement and the development of elite cultivars of *L. brownii* var. *viridulum*.

## Materials and methods

### Plant materials

The *L. brownii* var. *viridulum* used in this study were sourced from Shaoyang and Xuefeng Mountain, and were named ly and xf respectively. Line ly was collected from Longhui County, Shaoyang City, Hunan Province (111° 00′ 05.4″ E, 27° 06′ 13.6″ N), representing the traditional local cultivar. Line xf originates from the Xuefeng Mountains Huaihua City, Hunan Province (110°24′59.2″ E, 27°20′57.3″ N) and was identified as *L. brownii* var. *viridulum* following single-plant selection. These materials were cultivated in the lily germplasm resource nursery in Beidouxi Town, Xupu County, Huaihua City, Hunan Province, China (110° 32′ 6.8175″ E, 27° 39′ 11.7432″ N). Bulb samples with three biological replicates (xf1, xf2, xf3; ly1, ly2, ly3) were collected for metabolomic and RNA-seq analyses.

### Determination of total flavonoid content

The total flavonoid content was determined using a plant flavonoid content assay kit (BC1330/BC1335, Solarbio, Beijing). The procedure involved adding 0.2 g of freeze-dried, ground lily powder to 2 mL of extraction solution, followed by ultrasonic disruption. The supernatant was then mixed sequentially with the kit’s reagents, vortexed, and incubated in a 37 °C water bath for 45 min. After centrifugation at 10,000 *g* for 10 min at room temperature, 200 µL of the supernatant was transferred to a 96-well plate. Absorbance was measured at 470 nm. The standard curve was prepared using the same procedure as the samples. Flavonoid content was calculated as mg/g dry weight using the formula: Flavonoid content (mg/g) = x/W, where x is the concentration derived from the standard curve and W is the sample weight.

### Metabolome analysis

To elucidate metabolic changes in the ly and xf, an untargeted metabolomic analysis was performed on six bulb samples using liquid chromatography-tandem mass spectrometry (LC-MS/MS) by Maiwei Metabolic Biotechnology Co., Ltd. (Wuhan, China). Differential metabolites were identified using Log_2_FC (fold change) thresholds^23^. Subsequently, metabolites with significant altered accumulation were classified asupregulated or downregulated through statistical analysis. Differentially accumulated flavonoids were then screened and systematically categorized based on their metabolic profiles.

### RNA-seq and differential expression analysis

RNA-seq was performed by Maiwei Metabolic Biotechnology Co., Ltd. (Wuhan, China). Total RNA was isolated from lily bulbs. RNA purity, concentration, and integrity were evaluated using a NanoDrop 2000 spectrophotometer (Thermo Scientific, Pittsburgh, PA, USA). High-quality RNA samples were used to construct cDNA libraries, which were sequenced on the Illumina NovaSeq 6000 platform to generate 150-bp paired-end reads. After removing sequencing adapter and low-quality data, clean reads were obtained and aligned with the assembled transcriptome. Gene expression levels were quantified using fragments per kilobase per million (FPKM) normalization. DEGs were identified using the DESeq package in R, applying thresholds of *p* < 0.05 and |fold change (FC)| ≥ 2^24^. KEGG pathway enrichment analysis was performed to determine the functional roles and biological pathways associated with the DEGs.

### K-means and KEGG analysis

To identify genes related to flavonoid synthesis, we used the K-means tool on BMKCloud to conduct a cluster analysis of the DEGs detected in dragon teeth and lilies. Additionally, Kyoto Encyclopedia of Genes and Genomes (KEGG) pathway^25^ enrichment analysis was conducted to identify relevant modules using ggplots on the BMK Cloud platform.

### RNA isolation and quantitative real-time PCR (qRT-PCR)

Total RNA was isolated from the samples using the MiniBEST Plant RNA Extraction Kit (TaKaRa, Japan) following the manufacturer’s protocol. RNA integrity and quality using standard techniques, the RNA was reverse-transcribed into cDNA using the PrimeScript™ RT Reagent Kit (TaKaRa, Japan), following the specified protocol to ensure high-fidelity transcription. Quantitative real-time PCR (qRT-PCR) was performed on a LightCycler 480 Real-Time PCR System (Roche, Germany) using Hieff UNICON^®^ Universal Blue qPCR SYBR Green Master Mix (YESEN, Shanghai) to quantify the relative mRNA expression of target genes. The specific primers used for each target gene are listed in Table S5. The PCR conditions were as follows: initial denaturation at 95 °C for 30 s, followed by 40 cycles of 95 °C for 3 s and 60 °C for 20 s. Notably, the relative expression levels of the target genes were calculated using the 2^–ΔΔCT^ method and normalized to that of the lily *actin* gene (internal reference)^6^. All experiments were performed in triplicate for reproducibility and accuracy.

### Construction of phylogenetic tree

Briefly, protein sequences of lilies and various other plant species were retrieved from the National Center for Biotechnology Information (NCBI) database (available at https://www.ncbi.nlm.nih.gov/) to construct a phylogenetic tree. Sequence alignments were performed using ClustalW (http://www.ebi.ac.uk/clustalw/). To elucidate evolutionary relationships among these sequences, a phylogenetic tree was constructed using MEGA 5.0 software with the neighbor-joining (NJ) method. Evolutionary distances were inferred and topology was generated with bootstrap analysis (1000 replicates)^26^.

### Statistical analysis

Analysis of variance (ANOVA) was performed using SPSS Statistics 27 software (IBM, Chicago, IL, USA). Heatmaps were generated using Tbtools. Correlation network diagrams and heatmaps were constructed by integrating transcriptomics and metabolomics data using the Metware Cloud platform (https://cloud.metware.cn/#/user/login). Data were expressed as means ± standard deviation (SD).

## Results

### Metabolic differences between the bulb of ly and xf

**Fig. 1:**
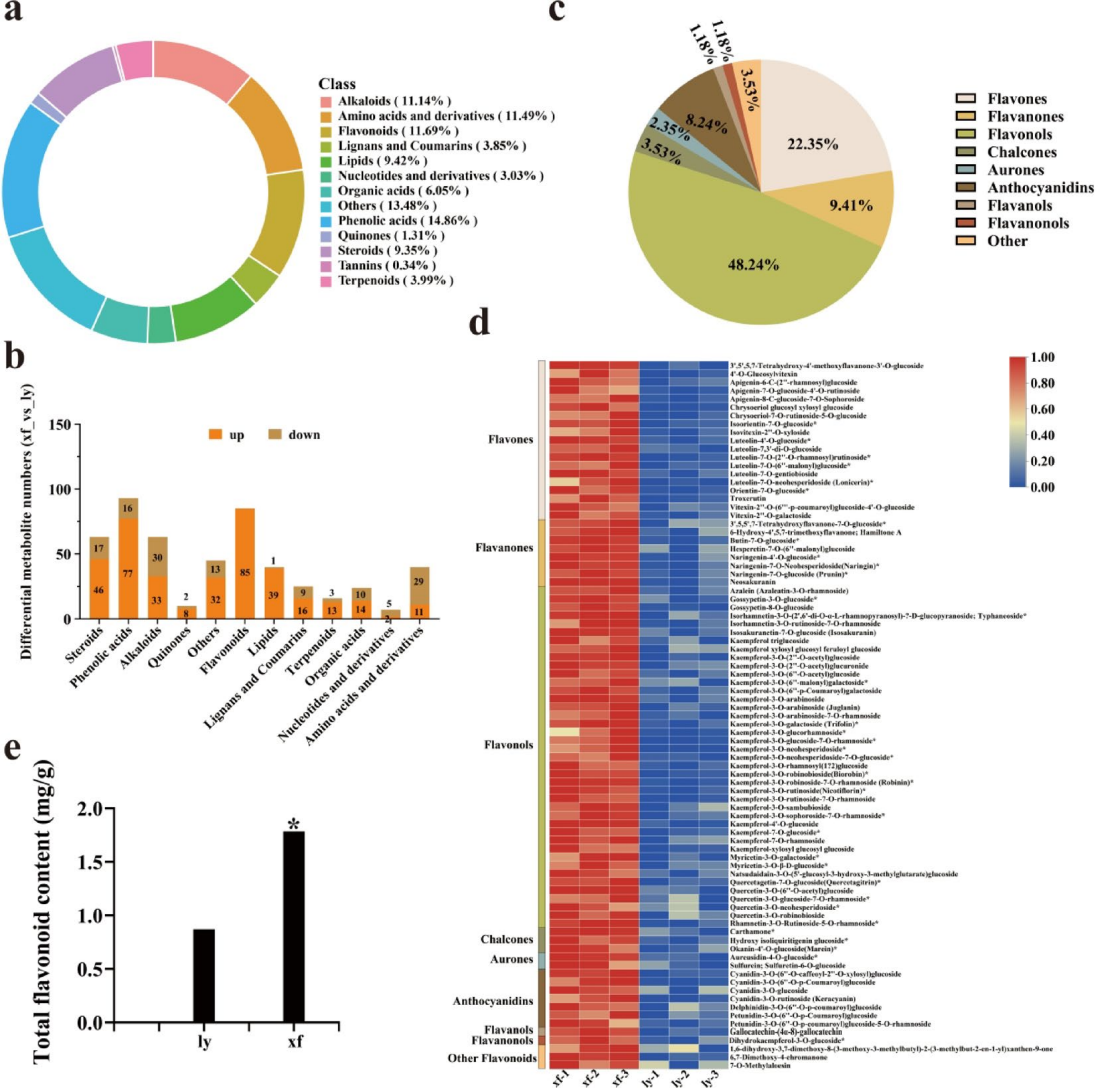
Metabolome analysis of differentially accumulated metabolites. (**a**) Metabolite classification ring diagram. Each color represents a class of metabolites, and the area of the color block indicates the proportion of the class. (**b**) Metabolites differentially accumulated in *L. brownii* var. *viridulum* bulbs from two distinct provenances (ly and xf). (**c**) Flavonoid compound classification pie chart. Each color represents a class of metabolites. (**d**) Heatmap of flavonoid accumulation in xf and ly. The color gradient from blue to red indicates an increasing concentration. (**e**) Total flavonoid content in the bulbs of ly and xf.

Comprehensive metabolite profiling was conducted on six lily samples using UPLC-MS/MS analysis. A total of 1454 metabolites were identified, with flavonoids and phenolic acids being the most dominant categories (Fig. 1a, Table S1). Comparative analysis was conducted to investigate metabolite composition differences between the ly and xf varieties of *L. brownii* var. *viridulum*. Notably, 352 upregulated and 125 downregulated metabolites were detected in the xf versus ly groups. All flavonoids in xf were upregulated compared to ly (Fig. 1b, Table S1). Moreover, 85 flavonoids were identified among the differentially expressed metabolites and categorized into several subclasses: flavones (22.35%), flavanones (9.41%), flavonols (48.24%), aurones (3.53%), anthocyanins (2.35%), flavanols (8.24%), flavanonols (1.18%), and others (1.18%) (Fig. 1c, Table S1). Furthermore, the significant enrichment of flavonoids in xf relative to ly was illustrated using a heatmap (Fig. 1d, Table S1). The total flavonoid content in xf bulbs was significantly higher than that in ly bulbs. (Fig. 1e). Collectively, these findings suggest that increased flavonoid accumulation in xf may enhance its pharmacological activity, indicating that these flavonoids represent the primary bioactive fractions in *L. brownii* var. *viridulum*.

### Comprehensive profiling of differentially expressed genes (DEGs) and transcription factors in ly and xf

**Fig. 2:**
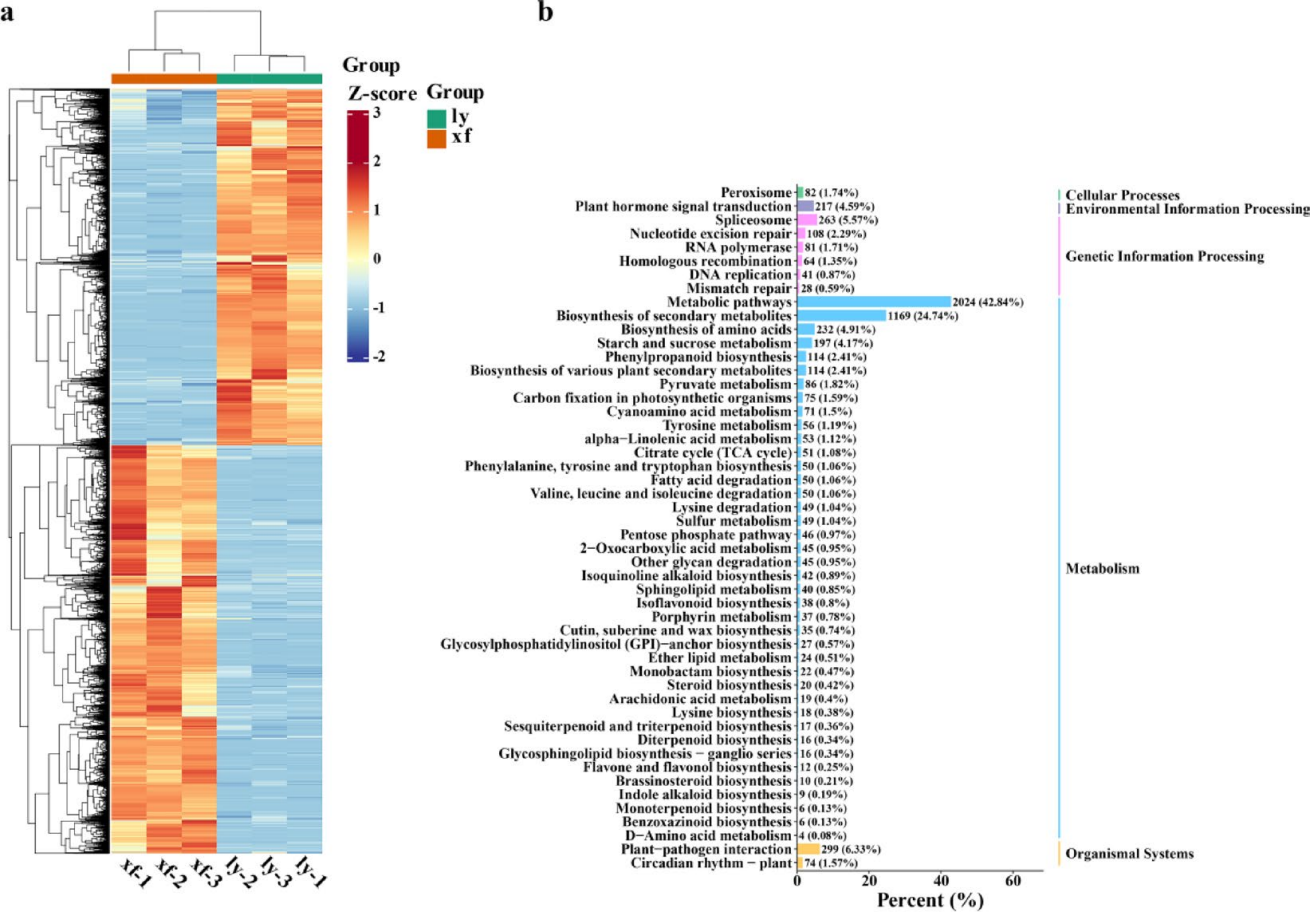
Differentially expressed genes (DEGs) in ly and Xuefeng varieties. (**a**) Heatmap analysis of DEGs in xf and ly. The colors in the heatmap represent Z-score values for gene expression levels, with red representing high expression and blue representing low expression. (**b**) Top 50 enriched pathways in the Kyoto Encyclopedia of Genes and Genomes (KEGG) database.

Total of 15,948 DEGs were identified in the bulb of ly compared with those in xf. Principal component analysis (PCA)analysis shows good reproducibility between samples, and there is a difference between ly and xf (Fig. S3). A clustering heatmap illustrates the gene expression trends of the genes in ly and xf (Fig. 2a, Table S2). Additionally, 737 differentially expressed transcription factors from 11 families were identified in both xf and ly (Fig. S3). Kyoto Encyclopedia of Genes and Genomes (KEGG) pathway enrichment analysis revealed that the DEGs were mainly enriched in flavonoid synthesis, including isoflavone, flavone, and flavonol biosynthesis (Fig. 2b).

### K-means analysis revealed DEGs related to flavonoids

**Fig. 3:**
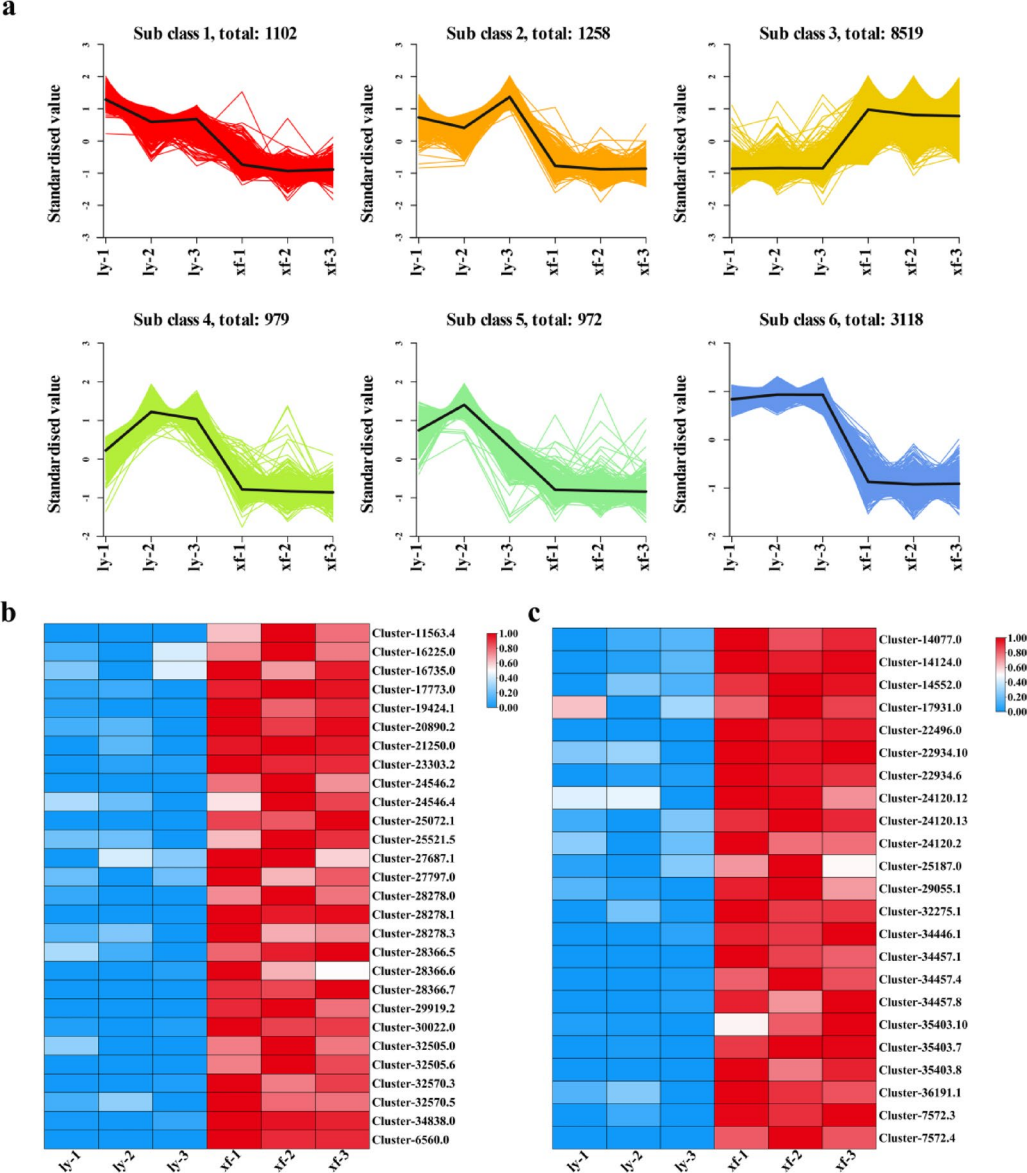
K-means clustering analysis presented the analytical results of the transcriptomes of ly and xf. (**a**) The K-means clustering analysis revealed the expression level of genes in ly and xf. (**b**) The expression of MYB transcription factor in the 3rd cluster in ly and xf. (**c**) The expression of bHLH transcription factor in the 3rd cluster in ly and xf. The color gradient from blue to red indicates an increase in expression level.

In this study categorized 15,948 DEGs into 6 distinct clusters using the k-means clustering algorithm. Further analysis of these clusters showed that the expression patterns of DEGs in the third cluster for both ly and xf aligned with flavonoid accumulation trends, indicating a potential association between these genes and flavonoid synthesis (Fig. 3a, Table S3). Among these genes, numerous transcription factors were identified. Notably, common transcription factors associated with flavonoid biosynthesis include 28 MYB and 23 bHLH genes, which exhibit significantly higher expression levels in xf compared to ly (Fig. 3b, c, Table S3).

### Structural genes involved in flavonoid synthesis

**Fig. 4:**
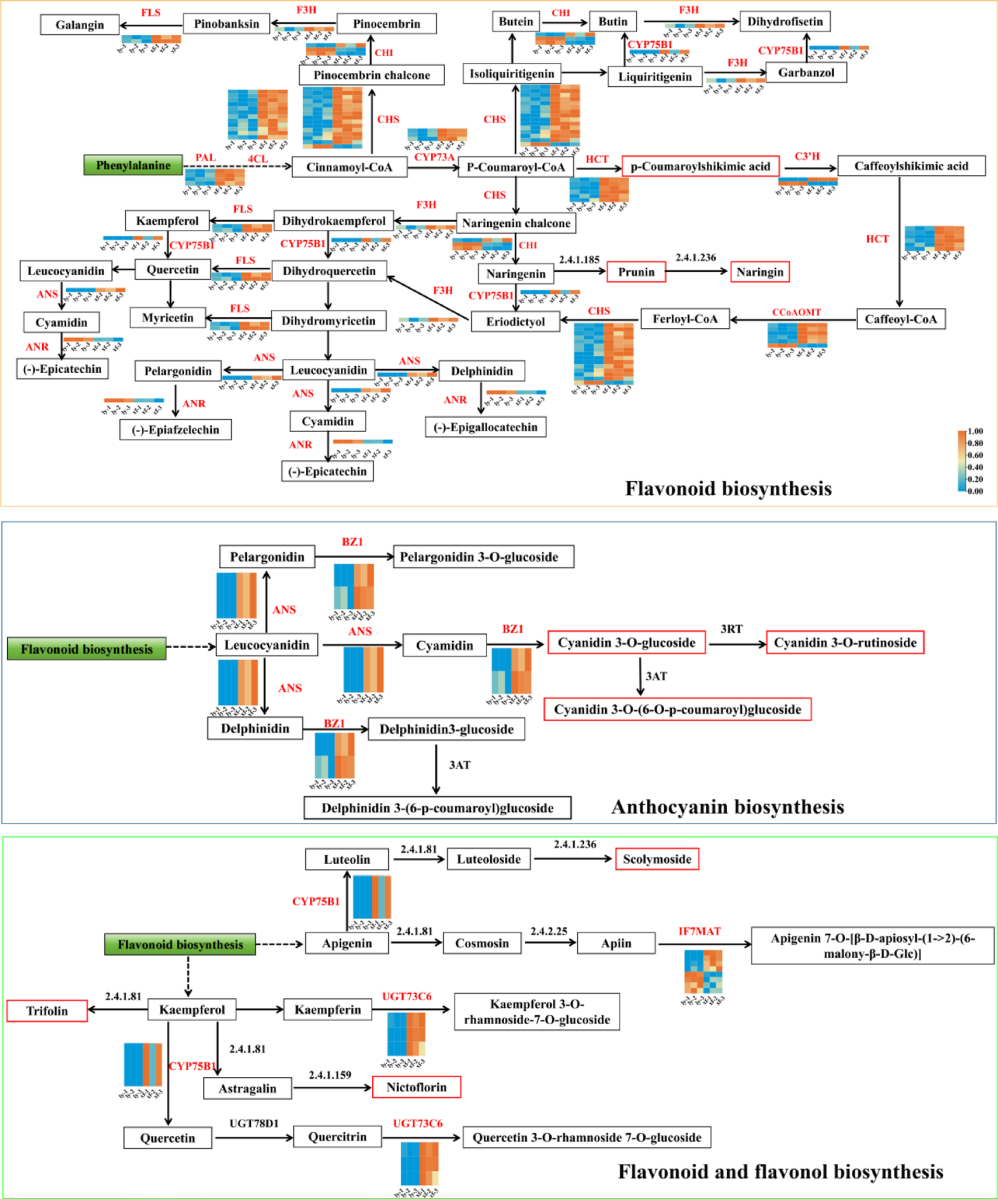
Reconstruction of the phenylpropanoid-flavonoid-anthocyanin-flavonoid and flavonol biosynthesis pathway in Misty blueberry. The heatmap in the above pathway represents the expression level of differentially expressed genes (DEGs). In the above heatmap, the darker color indicates a higher level of gene expression. The boxes represent metabolites and the red boxes indicate significantly accumulated flavonoids in *L. brownii var. viridulum.* FLS, flavonol synthase; F3H, naringenin 3-dioxygenase; CHS, chalcone synthase; PAL, phenylalanine ammonia-lyase; 4CL, 4-coumarate–CoA ligase; C3′H, 5-*O*-(4-coumaroyl)-D-quinate 3′-monooxygenase; FLS, flavonol synthase; ANS, anthocyanidin synthase; HCT, shikimate *O*-hydroxycinnamoyltransferase; CYP73A, trans-cinnamate 4-monooxygenase; CYP75B1, flavonoid 3′-monooxygenase; *EC:5.5.1.6*, chalcone isomerase (CHI); E2.1.1.104, caffeoyl-CoA *O*-methyltransferase (CCoAOMT); *ANR*, anthocyanidin reductase; UGT73C6, flavonol-3-*O*-L-rhamnoside-7-*O*-glucosyltransferase; IF7MAT, isoflavone 7-*O*-glucoside-6″-*O*-malonyltransferase.

**Fig. 5:**
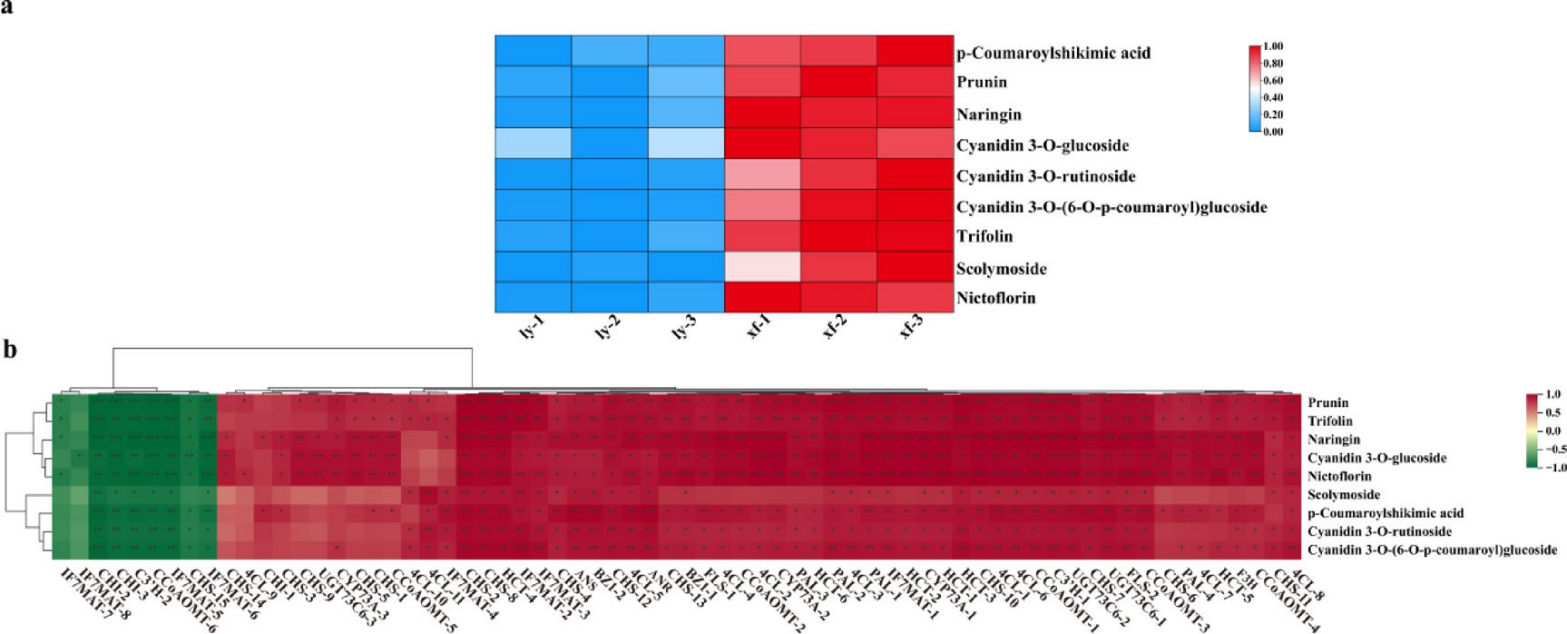
Screening of key candidate genes involved in flavonoid biosynthesis. (**a**) Heatmap analysis of differential metabolites in the flavonoid biosynthetic pathway. (**b**) Correlation analysis between differentially accumulated metabolites and differentially expressed genes in the flavonoid biosynthetic pathway. *P < 0.05, **: *P* < 0.01, ***P < 0.001. A negative correlation is indicated by green, while a positive correlation is represented by red. The intensity of the color corresponds to the degree of correlation, with darker shades indicating stronger correlations.

Based on the KEGG pathways related to flavonoid biosynthesis (ko00940, ko00941, ko00942, ko00943 and ko00944), we constructed a regulatory network that integrates differentially accumulated flavonoids with differentially expressed genes in xf versus ly. As shown in Figs. 4 and 5a and p-coumaroylshikimic acid, prunin, naringin, cyanidin 3-*O*-glucoside, cyanidin 3-*O*-rutinoside, cyanidin 3-*O*-(6-*O*-p-coumaroyl)glucoside, trifolin, scolymoside and nictoflorin were all significantly more abundant in xf. Additionally, 59 differentially expressed enzyme genes were identified, and the majority of enzymatic genes exhibited expression patterns congruent with the accumulation of their downstream flavonoids, including *FLS*,* F3H*,* CYP75B1*,* PAL*,* 4CL*,* CYP73A*,* HCT*,* C3′H*,* CHS*,* ANS*,* ANR*,* BZ1*,* UGT73C6*, and *IF7MAT*. Pearson correlation analysis between the relative abundances of differential flavonoids and the FPKM values of DEGs shown in Figs. 4 and 5a and p-coumaroylshikimic acid, prunin, naringin, cyanidin 3-*O*-glucoside, cyanidin 3-*O*-rutinoside, cyanidin 3-*O*-(6-*O*-p-coumaroyl)glucoside, trifolin, scolymoside, and nictoflorin were all significantly more abundant in xf. Additionally, 59 differentially expressed enzyme genes were identified, with most exhibiting expression patterns consistent with the accumulation of their downstream flavonoids, including *FLS*, *F3H*, *CYP75B1*, *PAL*, *4CL*, *CYP73A*, *HCT*, *C3′H*, *CHS*, *ANS*, *ANR*, *BZ1*, *UGT73C6*, and *IF7MAT*. Pearson correlation analysis between the relative abundances of differential flavonoids and the FPKM values of differentially expressed genes showed that most of these genes were significantly positively correlated with their corresponding metabolites, while nine genes displayed significant negative correlations. (Fig. 5b). Collectively, these findings indicate that the markedly higher flavonoid content in xf relative to ly is likely attributable to the regulatory effects of these genes.

### Transcription factors involved in the synthesis of flavonoids

**Fig. 6:**
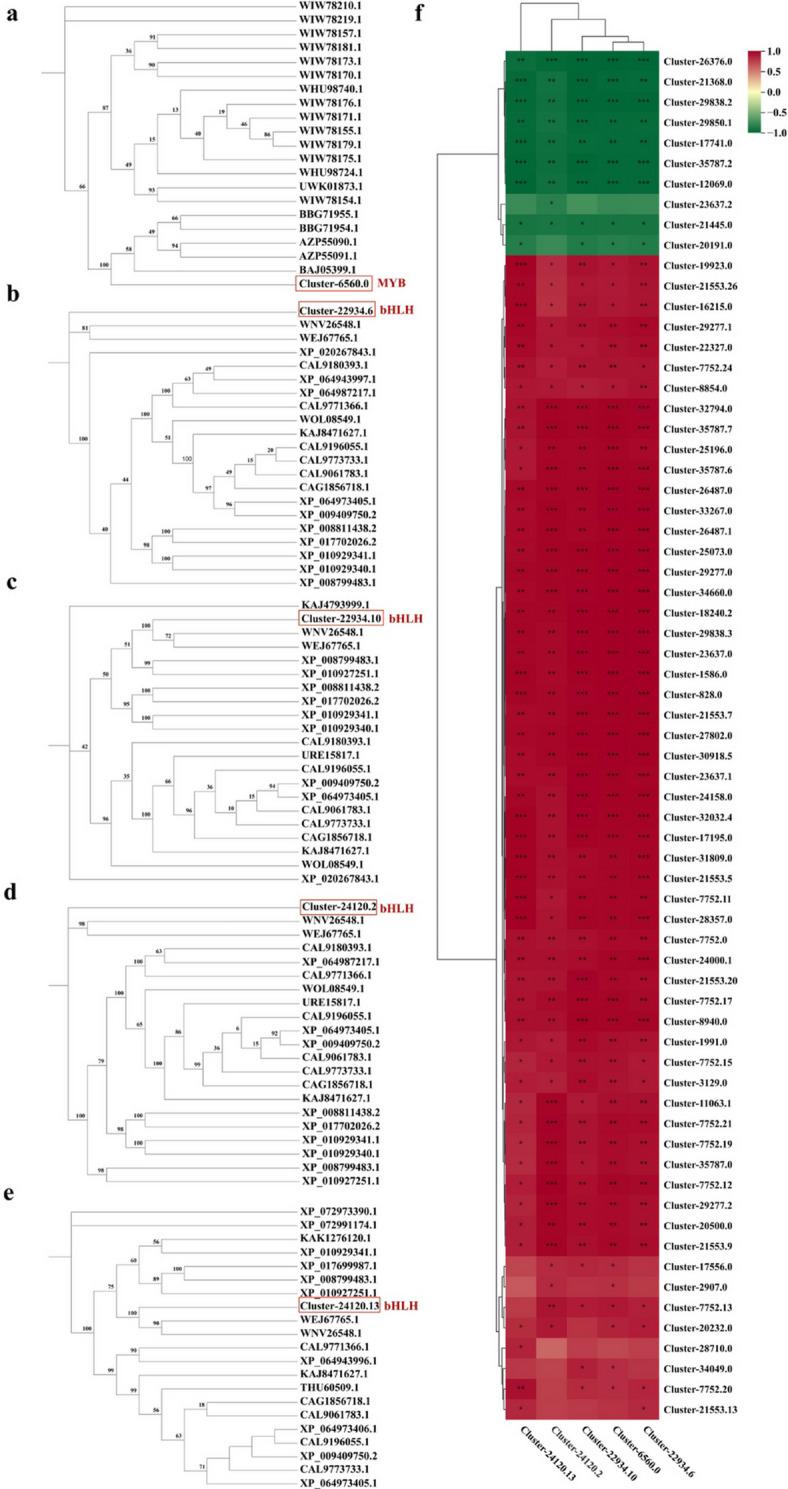
Phylogenetic analysis of MYB and bHLH transcription factors related to flavonoid synthesis and their correlation with functional genes. (**a**) Phylogenetic tree analysis of MYB protein (Cluster-6560.0) was performedand. (**b**–**e**) Phylogenetic tree analysis of bHLH proteins (Cluster-22934.6, Cluster-22934.10, Cluster-24120.2, and Cluster-24120.13) were performed. The top 20 sequence alignment results from NCBI were selected for constructing the phylogenetic tree (The protein sequence is detailed in Additional file 2). (**f**) Correlation heatmap analysis of MYB and bHLH transcription factors associated with flavonoid synthesis and their corresponding structural genes in the pathway was conducted. Cluster-6560.0, MYB transcription factor. Cluster-22934.6, Cluster-22934.10, Cluster-24120.2, and Cluster-24120.13, bHLH transcription factors. *FLS*: *Cluster-25196.0* and *Cluster-24158.0*. *F3H*: *Cluster-20500.0*. *CHI*: *Cluster-17556.0*, *Cluster-21368.0*, and *Cluster-17741.0*. *CYP75B1*: *Cluster-2907.0*. *CHS*: *Cluster-7752.19*, *Cluster-32032.4*, *Cluster-7752.13*, *Cluster-7752.15*, *Cluster-7752.21*, *Cluster-7752.12*, *Cluster-7752.17*, *Cluster-7752.11*, *Cluster-11063.1*, *Cluster-1586.0*, *Cluster-7752.20*, *Cluster-20232.0*, and *Cluster-21445.0*. *PAL*: *Cluster-31809.0*, *Cluster-29277.1*, *Cluster-29277.2*. *4CL*: *Cluster-21553.7*, *Cluster-21553.5*, *Cluster-32794.0*, *Cluster-21553.20*, *Cluster-27802.0*, *Cluster-21553.9*, *Cluster-21553.26*, *Cluster-21553.13*, *Cluster-28710.0*, *Cluster-21553.22*, *Cluster-21553.6*. *CYP73A*: *Cluster-26487.1*, *Cluster-8854.0*. *HCT*: *Cluster-25073.0*, *Cluster-34660.0*, *Cluster-28357.0*, *Cluster-17195.0*, *Cluster-24000.1*, *Cluster-22327.0*. *C3′H*: *Cluster-29838.3*, *Cluster-29838.2*. *CCoAOMT*: *Cluster-18240.2*, *Cluster-35787.7*, *Cluster-30918.5*, *Cluster-35787.6*, *Cluster-35787.2*. *ANS*: Cluster-5836.0. *ANR*: *Cluster-1991.0*. *BZ1*: *Cluster-8940.0*, *Cluster-5836.0*. *IF7MAT*: *Cluster-3129.0*, *Cluster-828.0*, *Cluster-19923.0*, *Cluster-16215.0*, *Cluster-34049.0*, *Cluster-12069.0*, *Cluster-26376.0*, *Cluster-20191.0*. *UGT73C6*: *Cluster-23637.2*, *Cluster-23637.1*, *Cluster-23637.0*. *CYP75B1*: *Cluster-2907.0*. *P < 0.05, **P < 0.01, ***P < 0.001. A negative correlation is indicated by green, while a positive correlation is represented by red. The intensity of the color corresponds to the degree of correlation, with darker shades indicating stronger correlations.

Transcription factors not only participate in various pathways that regulate plant growth and development but can also regulate the expression of specific genes in the flavonoid biosynthetic pathway. In this study, we identified 64 MYB and 44 bHLH transcription factors related to flavonoid synthesis in the third category. Among them, the expression levels of 28 MYB and 23 bHLH genes were significantly higher in xf than in ly (Table S4). Based on the results of the previous k-means clustering analysis, we selected candidate MYB and bHLH transcription factors associated with flavonoid biosynthesis from the third cluster and performed phylogenetic tree analysis. Phylogenetic analysis revealed that the MYB transcription factor Cluster-6560.0 and the bHLH transcription factors Cluster-22934.6, Cluster-22934.10, Cluster-24120.2, and Cluster-24120.13 predominantly regulated anthocyanin biosynthesis (Fig. 6a–e). Transcriptional correlation analysis within the flavonoid biosynthesis pathway indicated that five structural genes (*Cluster-26376.0*/*IF7MAT*, *Cluster-29838.2*/*C3′H*, *Cluster-29850.1*/*ANR*, *Cluster-17741.0*/*E5.5.1.6*, and *Cluster-35787.2*/*E2.1.1.104*) showed significant negative correlations with the transcription factors (*p* < 0.01). Additionally, three structural genes (*Cluster-23637.2*/*UGT73C6*, *Cluster-21445.0*/*CHS*, and *Cluster-20191.0*/*IF7MAT*) exhibited relatively weak negative correlations with the transcription factors. Notably, the three structural genes demonstrated consistent expression patterns in the ly and xf varieties. In contrast, most of the remaining genes showed strong positive correlations with the transcription factors, with most associations being statistically significant (*p* < 0.01) (Fig. 6f). This result also showed that MYB transcription factor Cluster-6560.0 and the bHLH transcription factors Cluster-22934.6, Cluster-22934.10, Cluster-24120.2, and Cluster-24120.13 may regulate anthocyanin synthesis by regulating the expression of *FLS*, *F3H*, *CHI*, *PAL*, *4CL*, *CYP73A*, *HCT*, and *C3′H* (Fig. 6f). Overall, the screened transcription factors were associated with flavonoid synthesis.

### Verification of the key enzyme genes for the synthesis of flavonoid compounds

**Fig. 7:**
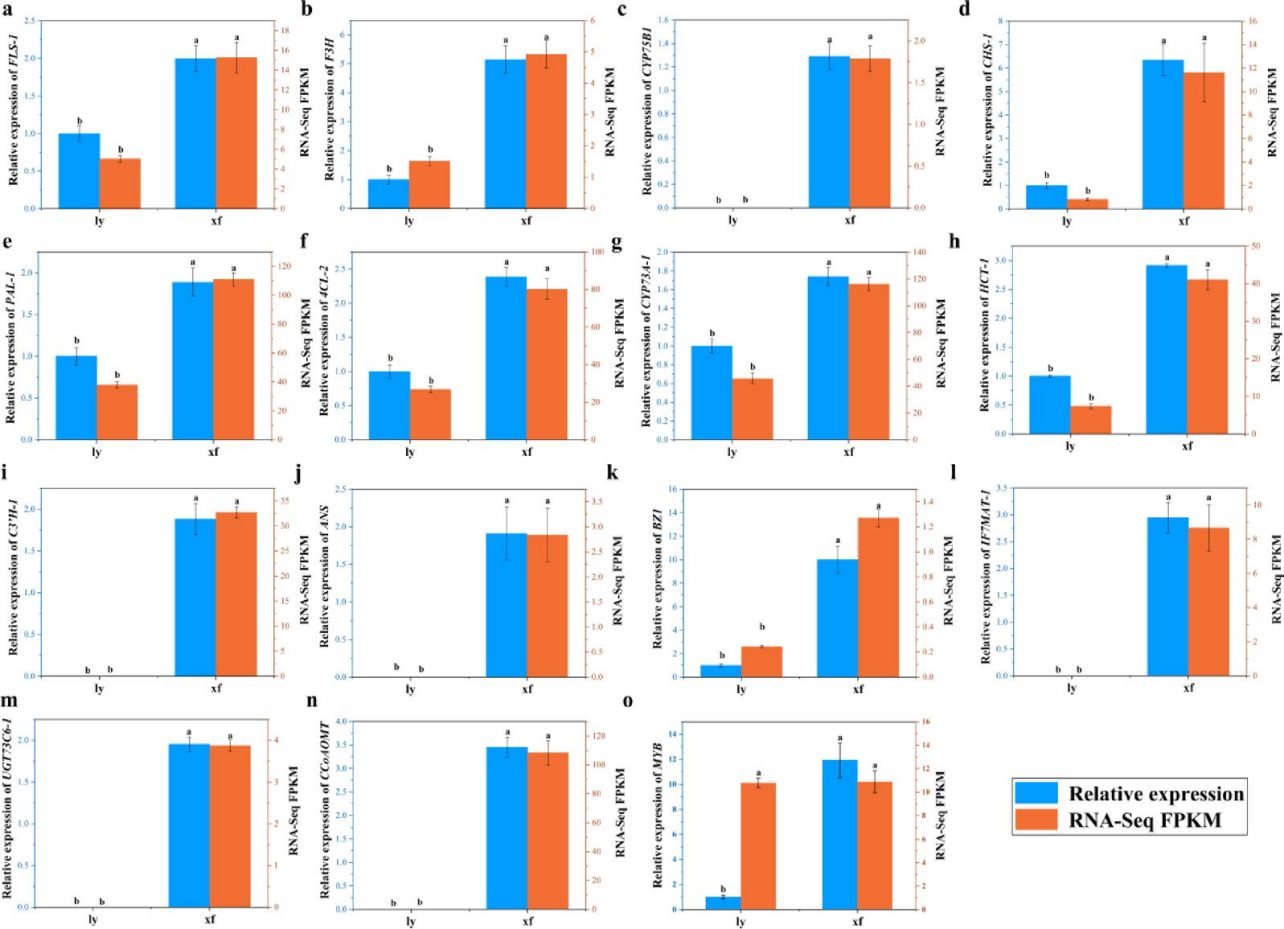
Transcriptome data were validated using qRT-PCR.

To characterize the expression patterns of genes associated with flavonoid biosynthesis, a comprehensive analysis was conducted on enzyme-coding genes in the flavonoid biosynthesis pathway of *L. brownii* var. *viridulum* (ly and xf). Transcriptomic profiling revealed that all 14 enzyme-coding genes had significantly higher expression levels in xf compared to ly. Furthermore, the MYB transcription factor (Cluster-6560.0), which is associated with anthocyanin synthesis, exhibited a corresponding expression pattern. Quantitative real-time PCR (qRT-PCR) results confirmed this expression trend, validating the reliability of the transcriptome data (Fig. 7).

## Discussion

Flavonoids serve as the primary antioxidants in plants and are the dominant specialized metabolites in Lilium^27^. Previous researchhas demonstrated that the medicinal-food cultivar ly (*L. brownii* var. *viridulum*) possesses lower flavonoid abundance and weaker antioxidant activity compared to *Lilium regale*^27^. In this study, comparative metabolomic profiling of two *L. brownii* var. *viridulum* cultivars (ly and xf) revealed that flavonoids were the most abundant phytochemical class and were markedly enriched in xf (Fig. 1), effectively offsetting the inherent flavonoid deficiency of ly. These findings highlight the potential of high-flavonoid cultivars like xf as sources of natural antioxidants for functional foods, cosmeceuticals, and nutraceuticals.

Flavonoid synthesis involves various biosynthetic pathways, including phenylalanine, flavonoid, anthocyanin, flavone, and flavonol pathways, along with numerous structural and regulatory genes^28^. To identify the candidate structural genes, we performed k-means clustering on DEGs. The results showed that the expression trend of the genes in cluster 3 aligned with the enrichment trend of flavonoids (Fig. 3). Integrated transcriptomic and metabolomic analyses further identified 14 enzyme-coding genes involved in flavonoid biosynthesis, namely *FLS*,* F3H*,* CYP75B1*,* PAL*,* 4CL*,* CYP73A*,* HCT*, *C3′H*,* CHS*,* ANS*,* ANR*,* BZ1*,* UGT73C6*, and *IF7MAT* (Figs. 4 and 5). Among them, 4CL and PAL have been identified as enzymes participating in phenylpropanoid synthesis in *L*. *davidii* var. unicolor and *L. lancifolium* Thunb. Additionally, ANS has been identified as a positive regulator of anthocyanins biosynthesis in the flower buds of *Lilium* “Siberia,” a finding consistent with our findings^29^. In this study, *ANS* positively regulates the downstream metabolites cyanidin 3-*O*-glucoside, cyanidin 3-*O*-rutinoside, and cyanidin 3-*O*-(6-*O*-p-coumaroyl)glucoside, all of which accumulate significantly in xf (Figs. 4 and 5). Furthermore, the remaining enzyme genes are involved in flavonoid synthesis in other species^30^.

Regulatory genes encode transcription factors or other cis-acting elements that can regulate the transcription of key genes in the biosynthetic pathway^15^. In flavonoid metabolism, the best-characterised TFs belong to the MYB, bHLH and WD40 families. These TFs can function independently or as part of a ternary MBW (MYB–bHLH–WD40) complex. The MBW complex demonstrates stronger trans-activation of flavonoid structural genes compared to any single TF1^31^. In black rice, the MBW complex consisting of OsMYB3, OsKala4 (bHLH) and OsTTG1 (WD40) activates anthocyanin biosynthesis^33,34^. In wheat, the R2R3-MYB factors TaMYB10-A1, -B1 and -D1 induce anthocyanin accumulation in the shoot apical meristem without a reported bHLH partner^35^. Similarly, the R2R3-MYB FtMYB31 in tobacco upregulates *FLS*, *F3H* and *CHS*, thereby enhancing rutin and total flavonol production^36^. OsKala4 and OsRc (bHLH factors) regulate black and red pericarp pigmentation in rice, respectively. Together with Rb and OsB2 (bHLH factors), they directly activate *F3H*, *DFR* and *ANS* to promote anthocyanin synthesis^32,35^. In this study, one MYB (Cluster-6560.0) and four bHLH TFs (Cluster-22934.6, Cluster-22934.10, Cluster-24120.2 and Cluster-24120.13) were differentially expressed in *L. brownii* var. *viridulum* bulbs and showed significant homology to known flavonoid regulators (Fig. 5). These findings indicate that both MYB and bHLH TFs are involved in anthocyanin and broader flavonoid biosynthesis in *L. brownii* var. *viridulum*. Future research will focus on determining whether the identified bHLH–MYB transcription factor complex contributes to flavonoid biosynthesis within lily bulbs.

Elucidating the regulatory mechanisms behind flavonoid divergence in *L. brownii* var. *viridulum* is critical for linking its chemical composition and biological efficacy, which will aid in the sustainable utilization of its medicinal and nutritional properties. In this study, integrated metabolomic and transcriptomic analyses identified candidate structural and regulatory genes associated with flavonoid biosynthesis. Future research should focus on functionally characterizing these genes and the elucidation of how transcription factors interact with structural genes to precisely regulate flavonoid accumulation.

## Supplementary Information

Below is the link to the electronic supplementary material.

Supplementary Material 1

## Data Availability

All data generated or analysed during this study are included in this published article and its supplementary information files.
